# Headache Onset Timing as a Predictor for Comorbid Conditions of Pediatric Primary Headache

**DOI:** 10.3390/medsci14010034

**Published:** 2026-01-08

**Authors:** Hideki Shimomura, Sachi Tokunaga, Eisuke Terasaki, Naoko Taniguchi, Yohei Taniguchi, Saeka Yoshitake, Masumi Okuda, Yasuhiro Takeshima

**Affiliations:** Department of Pediatrics, School of Medicine, Hyogo Medical University, 1-1 Mukogawa, Nishinomiya 663-8501, Hyogo, Japan

**Keywords:** headache onset timing, pediatric headache, migraine, tension-type headache, comorbid condition

## Abstract

**Background/Objectives**: Pediatric patients with primary headaches frequently exhibit diverse comorbid conditions, often rendering their headaches intractable. Early identification of and intervention for comorbid conditions are crucial for improving prognosis, yet remain challenging. We hypothesized that headache onset timing can predict the presence of these comorbid conditions. **Methods**: Headache onset timing of 106 pediatric patients (aged 6–17 [median: 13] years) with migraine or tension-type headache and associated comorbidities, including neurodevelopmental and sleep disorders, orthostatic intolerance (OI), and psychosocial factors, was retrospectively analyzed. **Results**: Headache onset timing was most frequent upon awakening (33.0%), followed by indeterminate (31.1%) and orthostatic (20.8%) onsets. OI (40.6%) and psychosocial factors (38.7%) were the most prevalent comorbid conditions. Psychosocial factors were most common in the awakening (62.9%) and indeterminate (27.3%) onset groups; OI predominated in the orthostatic group (77.3%). Multivariate analysis revealed that psychosocial factors were a significant risk factor for awakening headache (odds ratio [OR]: 4.59, 95% confidence interval [CI]: 1.80–11.71). OI was a risk factor for orthostatic onset headache (OR: 7.18, 95% CI: 1.92–26.87) and inversely associated with indeterminate headache (OR: 0.15, 95% CI: 0.04–0.54). **Conclusions**: Our findings suggest that detailed classification of headache onset timing can predict potential risks of specific comorbid conditions in pediatric patients.

## 1. Introduction

Headache is the most common complaint among children and adolescents, significantly affecting their quality of life and social functioning, as well as those of their families [1,2,3].

Children and adolescents with migraine or tension-type headache often experience various comorbid conditions, including sleep disorders, allergies, epilepsy, obesity, psychiatric and neurodevelopmental disorders, and orthostatic intolerance (OI), which make these headaches intractable [4,5,6]. Early identification of comorbid conditions is crucial for improving headache prognosis. However, determining which comorbid condition requires intervention is challenging because patients may present with multiple conditions that can be difficult to identify.

The International Classification of Headache Disorders, 3rd Edition (ICHD-3) [7], serves as the basis for headache diagnoses. This classification system broadly divides headaches into primary and secondary headaches. Primary headaches have no known cause and comprise 13 distinct types, including migraine and tension-type headache. Secondary headaches have an underlying cause; the ICHD-3 diagnostic criteria state that “a secondary headache can be definitively diagnosed only when solid evidence exists from published scientific studies.” Consequently, several medical conditions are not listed in the ICHD-3 because of insufficient solid evidence despite epidemiological reports indicating a propensity for headaches. Therefore, clinicians should diagnose these headaches as “a primary headache with a comorbid condition.” However, standard treatment for primary headache alone is not efficacious for these headaches [8]. Early identification of comorbid conditions is essential to provide proper treatment; however, given the nature of the pediatric population, these patients are often unaware of signs indicating comorbid conditions. As conducting all diagnostic tests for each comorbid condition is not feasible, clinicians should be able to predict the existence of comorbid conditions based on headache characteristics.

Headache onset timing represents a clinically important factor. However, a paucity of existing literature provides a comprehensive overview of headache onset timing. Morning headaches are sometimes indicative of a secondary headache. Conditions such as brain tumors, sleep disorders, and OI can cause these headaches [9,10,11]. Primary headache, especially migraine, can also occur in the morning [12,13]. Studies have suggested a correlation between sleep disturbances and stress [12,13]; however, the specific risk factors for morning headache remain unclear. Moreover, these studies focused on adult participants, with few reporting on children.

In this study, we analyzed headache onset timing in children with migraine or tension-type headache and comorbid conditions. We focused on headache onset timing, especially those that occurred during morning hours, and further classified them based on their occurrences at awakening, after standing up, or later in the morning. We hypothesized that detailed classifications of morning headache in children with migraine or tension-type headache might help predict the presence of comorbid conditions.

## 2. Methods

### 2.1. Patients

A retrospective analysis was conducted using the medical records of pediatric patients who were diagnosed with migraine or tension-type headache in the child neurology clinic at our hospital between 2021 and 2023. Our hospital is an urban university-affiliated hospital, designated as a tertiary referral center for the management of headache disorders and OI. Headache management was performed by a physician who was board-certified in both Pediatric Neurology and Headache Medicine.

The study participants were patients who presented with headache as their chief complaint. Headache diagnoses were made according to the ICHD-3. Patients with secondary headache disorders, including those attributed to traumatic brain injury, concussion, or other intracranial pathologies, were excluded from the study. We strictly followed the ICHD-3 criteria to ensure that only primary headache disorders were analyzed, thereby eliminating the influence of post-traumatic factors. To exclude secondary headaches, all patients underwent comprehensive blood tests, including complete blood count, liver and renal function tests, and serum iron, zinc, ferritin, and thyroid function measurements. Neuroimaging studies were performed at the discretion of the attending physician based on individual clinical symptoms, without adherence to rigid predefined criteria. A migraine diagnosis included migraine with and without aura.

Subject identification and data extraction were performed by two board-certified Pediatric Neurologists who were also certified in Headache Medicine. These data were collected retrospectively from electronic medical records. Data were collected on patient age at initial consultation, sex, headache frequency, and comorbid conditions, including neurodevelopmental disorders, OI, sleep disorders, and psychosocial factors. Monthly headache frequency was calculated by multiplying the weekly frequency by four.

Headache onset timing was initially recorded by the patients using a questionnaire regarding their average onset patterns during their first visit. These data were subsequently verified in direct interviews with the patients and their guardians. Headache onset timing was meticulously documented and subsequently categorized into five distinct classifications: morning, daytime, evening, nighttime, and indeterminate. The indeterminate category was reserved for cases where a precise onset time could not be ascertained. For enhanced granularity, the morning category was further subdivided into three specific subcategories. Headaches classified as awakening commenced precisely upon the patient’s awakening. Orthostatic onset headache was defined as a type of headache that manifests during the process of standing up from a lying position just after awakening or immediately after arising from bed, and midmorning headache was defined as an episode with headache symptoms manifesting within 1 h after the patient stands up and prior to daytime onset.

Regarding comorbid conditions, neurodevelopmental disorder diagnoses, including attention deficit hyperactivity and autism spectrum disorders, were determined by a child neurologist based on the criteria of the Diagnostic and Statistical Manual of Mental Disorders, Fifth Edition [14]. An OI diagnosis was determined based on at least two of the following five symptoms being present at least once a week and persisting for at least 6 months: susceptibility to vertigo and dizziness upon standing; tendency to faint when standing (which in severe cases may cause falls); nausea when taking a hot bath or in unpleasant situations; palpitation and/or dyspnea after mild exercise; and difficulty getting out of bed. These criteria were based on the Japanese diagnostic criteria for orthostatic dysregulation [15]. Sleep disorder was diagnosed following the ICHD-3 criteria [16]. Sleep-related data were collected via questionnaires, which included the following parameters: bedtime, sleep onset latency, awakening time, presence of nocturnal awakenings, and daytime sleepiness. Psychosocial factors were identified based on psychotherapy administration rather than patients merely reporting stress. During the first consultation, psychosocial factors were initially screened using questionnaires and a validated checklist for psychosomatic diseases in Japanese [17]. For patients whose headache symptoms did not improve within a 3-month period without psychotherapy, those factors were re-evaluated. A definitive diagnosis of comorbid psychosocial factors was established only if the symptoms subsequently improved following psychotherapy specifically targeting those factors.

This study was conducted in accordance with the Declaration of Helsinki and other relevant national regulations and guidelines. The requirement for informed consent was waived owing to the retrospective nature of the study. The study design was approved by the Review Board of Hyogo Medical University, Hyogo, Japan (approval number 4135, approval date: 21 July 2022).

### 2.2. Statistical Analyses

All statistical analyses were conducted using JMP 18 (SAS Institute Inc., Cary, NC, USA). The frequency of comorbid conditions at each headache onset timing and comparisons of morning headache prevalence between patients without comorbid conditions and each comorbid group were analyzed using Fisher’s exact test. Kendall’s rank correlation coefficient was used to determine overlap among comorbid conditions. A multiple logistic regression analysis was performed to assess factors that affect headache onset timing. The following covariates were incorporated into the analysis: the binary presence (yes/no) of four specific comorbid conditions—OI, neurodevelopmental disorders, sleep disorders, and psychosocial factors—along with the headache diagnosis (migraine or tension-type headache) and the basic demographic data of sex and age. In addition, headache frequency was incorporated into the multivariate model to account for the potential confounding effects of chronicity. Given the significant ceiling effect in its distribution, frequency was dichotomized into “daily” (28 days/month) and “non-daily” (<28 days/month) to maintain statistical stability and minimize over-adjustment bias. Furthermore, the small subgroups (midmorning, daytime, evening, and nighttime onsets) were combined into a single reference group to ensure sufficient statistical power for the regression analysis. To rigorously justify the model’s structural integrity amidst the clinical complexity of a cohort in which nearly half exhibited multimorbidity, we assessed multicollinearity using variance inflation factors (VIF). A stringent threshold was applied, and the variables with high collinearity were scrutinized to ensure independence of the covariates. Model fit was evaluated using comprehensive diagnostics, including the likelihood ratio test, McFadden’s pseudo-*R*^2^ (*R*^2^(*U*)), and the Akaike information criterion. Statistical Significance was set at *p* < 0.05.

## 3. Results

Table 1 lists the demographic characteristics of the 106 patients (41 male and 65 female, aged 6–17 [median 13] years). Migraine affected 90 patients (84.9%), whereas tension-type headache affected 16 patients (15.1%).

Headache onset timing was classified into five categories: morning, daytime, evening, nighttime, and indeterminate. The morning category was further divided into three subcategories: awakening, orthostatic, and midmorning.

Overall, 35 patients (33.0%) had headache onset upon awakening, 33 patients (31.1%) had indeterminate onset, and 22 patients (20.8%) had orthostatic onset. Regarding comorbid conditions, 72 patients (67.9%) had at least one comorbid condition. OI emerged as the most prevalent comorbid condition, affecting 43 patients (40.6%), followed by psychosocial factors affecting 41 patients (38.7%), sleep disorders affecting 38 patients (35.8%), and neurodevelopmental disorders affecting 16 patients (15.1%). Thirty-four patients (32.1%) did not exhibit comorbid conditions, and 45 patients (42.5%) had two or more comorbid conditions.

The analysis of morning headache prevalence among patients with and without each comorbid condition revealed that 9 of 34 patients (26.5%) without comorbid conditions, 12 of 16 patients (75.0%) with neurodevelopmental disorder, 38 of 43 patients (88.4%) with OI, 29 of 38 patients (76.3%) with sleep disorder, and 30 of 41 patients (73.2%) with psychosocial factors experienced morning headache. Regarding headache frequency, the study population showed a highly skewed distribution; 63 of 106 patients (59.4%) reported daily headaches (28 days/month). Morning headache was significantly more frequent among all the patients with comorbid conditions (neurodevelopmental disorder, *p* = 0.002; OI, *p* < 0.001; sleep disorder, *p* < 0.001; psychosocial factors, *p* < 0.001).

The high incidence of overlapping comorbid conditions led us to analyze these trends (Table 2). We found that sleep disorders significantly co-occurred with OI (τ = 0.384, *p* < 0.0001) and psychosocial factors (τ = 0.2142, *p* = 0.0282). In the awakening group, psychosocial factors predominated (62.9%), followed by OI (57.1%) and sleep disorders (51.4%). In the orthostatic group, OI predominated (77.3%), followed by sleep disorders (45.5%) and psychosocial factors (27.3%). In the indeterminate group, psychosocial factors predominated (27.3%), followed by sleep disorder (15.2%) and OI (12.1%) (Table 3).

**Figure 1:**
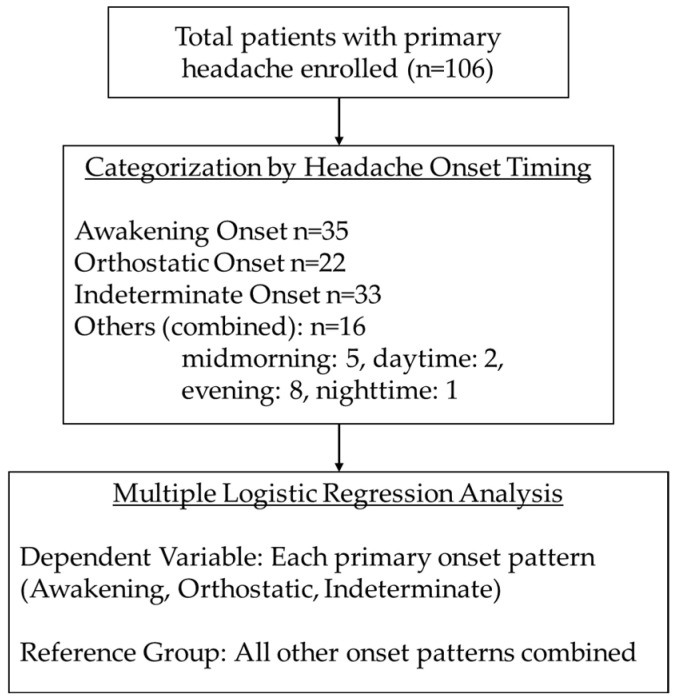
Flowchart of patient categorization and the structure for the multiple logistic regression models.

To elucidate the independent associations between specific headache onset timings and comorbid conditions, a series of multiple logistic regression analyses was conducted. The process of patient categorization and the regression model structures are illustrated in Figure 1. For each analysis, the target onset pattern was compared against a reference group consisting of all other onset patterns to maintain the total sample size (*n* = 106) and ensure statistical stability. The constructed model for awakening headache onset exhibited a significant fit to the observed data (likelihood ratio χ^2^ = 22.95, *p* = 0.0034), indicating its robust capacity to differentiate between clinical outcomes. In line with the model’s diagnostic integrity, McFadden’s pseudo-R^2^ was 0.171, and the Akaike information criterion was 131.4. To assess multicollinearity, the VIF was calculated for all covariates, with a maximum value of 1.64, which confirmed no significant issues. To maintain the total sample size (*n* = 106) and ensure statistical stability, the small subgroups midmorning (*n* = 5), daytime (*n* = 2), evening (*n* = 8), and nighttime (*n* = 1) were combined into a single reference group (all other onset patterns) for each multiple regression analysis. Consequently, the analysis focused on the three primary headache onset patterns: awakening (*n* = 35), orthostatic (*n* = 22), and indeterminate (*n* = 33). Detailed estimates for each parameter, represented by odds ratios (ORs) and their corresponding 95% confidence intervals (CIs), are summarized in Table 3. The multiple logistic regression analysis of comorbid conditions as risk factors for headache onset timing indicated that psychosocial factors had a significant effect on awakening headache development (OR: 4.59, 95% CI: 1.80–11.71). OI significantly affected orthostatic onset headache development (OR: 7.18, 95% CI: 1.92–26.87), whereas it had a reduced effect on indeterminate headache development (OR: 0.15, 95% CI: 0.04–0.54). The ORs were interpreted as indicators for the association strength between specific headache onset timings and comorbid conditions. Specifically, a high OR for psychosocial factors in awakening headache and for OI in orthostatic onset headache reflects a robust clinical association, whereas an OR below 1.0 (OI in indeterminate headache) suggests a significant inverse association.

## 4. Discussion

In this study, we determined a relationship between headache onset timing and comorbid conditions in pediatric patients. Overall, 67.9% of the patients diagnosed with migraine or tension-type headache had a comorbid condition, with OI being the most prevalent. Morning headache prevalence was significantly higher in patients with comorbid conditions than in those without. The comparative analysis showed the risk of psychosocial factors was highest for awakening headache, whereas the risk of OI was highest for orthostatic onset headache. These findings suggest that detailed classifications of headache onset timing can be used as predictors for the potential risks of specific comorbid conditions.

Morning headaches often indicate a secondary headache caused by conditions such as brain tumors, sleep disorders, and orthostatic hypotension [9,10,11]. The ICHD-3 lists morning headache as a feature of several conditions, including “headache attributed to dural arteriovenous fistula,” “headache attributed to intracranial neoplasm,” “headache attributed to low cerebrospinal fluid pressure,” “sleep apnea headache,” and “head and/or neck pain attributed to orthostatic (postural) hypotension.” When classified based on headache onset timing, “sleep apnea headache” occurs upon awakening, whereas “headache attributed to low cerebrospinal fluid pressure” and “head and/or neck pain attributed to orthostatic (postural) hypotension” occur when standing. However, the onset timing of other headache types remains unclear. Brain tumor-related headache, which is described in the ICHD-3 as “headache attributed to intracranial neoplasia,” had been traditionally perceived to worsen in the morning or when lying down; however, recent reports indicate that adult patients do not present with such headaches [18]. In contrast, nocturnal and morning headaches are more common among pediatric patients with brain tumors [10]. The ICHD-3 states that “headache attributed to dural arteriovenous fistula” primarily occurs in the morning, yet studies on this condition are limited [19]. Further research is needed to clarify these associations. According to the ICHD-3, “head and/or neck pain attributed to orthostatic (postural) hypotension” manifests as an orthostatic headache and is linked to conditions such as spinal cord injury, pure autonomic failure, and multiple system atrophy. Although “head and/or neck pain attributed to orthostatic (postural) hypotension” may appear similar, childhood OI in the present study represented a different pathophysiology.

Notably, “orthostatic onset headache” as defined in this study must be distinguished from the ICHD-3 diagnostic entity of “orthostatic headache” (ICHD-3 7.2.1), which typically suggests low cerebrospinal fluid pressure. In our cohort, “orthostatic onset headache” referred strictly to the temporal pattern of headache initiation during physical movement. Although secondary causes were clinically excluded during the initial workup, our findings should be interpreted specifically within the context of onset timing rather than as a distinct headache pathology. From a clinical perspective, it is essential to consider the differential diagnosis of post-traumatic headache resulting from traumatic brain injury or concussion, as outlined in recent clinical guidelines [20]. Post-traumatic headache can present phenotypes similar to migraine or tension-type headache; however, it often involves complex symptoms such as balance issues and cognitive difficulties that were not observed in our primary headache cohort. This distinction suggests that the correlations between headache onset timing and comorbidities revealed in this study are characteristic features of primary headache disorders, independent of traumatic influences.

Comorbid conditions in children with migraine or tension-type headache present a significant challenge [4,5]. A comprehensive cross-sectional study investigated the correlations between headache development and various lifestyle factors and comorbid conditions [5]. Among these, sleep habits, allergies, psychiatric disorders and medications, and vitamin intake emerged as associated variables. However, determining the comorbid conditions that contribute to headache onset or exacerbation during an initial visit remains challenging. The early identification of comorbid conditions requires careful evaluation of headache characteristics; few published studies have addressed these conditions. The present study demonstrated an elevated risk of comorbid OI in children with orthostatic onset headache and increased psychosocial factors in those with headaches occurring upon awakening. Conversely, the risk for comorbid OI was lower for patients with headaches with an indeterminate onset. These results may aid in the earlier identification of comorbid conditions.

A considerable number of studies have indicated a high coexistence of neurodevelopmental disorders and recurrent headaches [21,22,23]. The etiology of comorbid headache in patients with neurodevelopmental disorders remains poorly understood; however, studies have suggested that monoamines, including dopamine, noradrenaline, and gamma-aminobutyric acid, may play a role [24,25]. Some research has suggested an association of other factors with sensory hypersensitivity resulting from neurodevelopmental disorders. Sullivan et al. [22] reported that children with autism spectrum disorders with comorbid migraine exhibit greater sensory hyper-responsiveness than those without migraine. Sensory hyper-responsiveness presents a major challenge for children with neurodevelopmental disorders and may contribute to sleep disturbances, stress, and difficulties with social adjustment, in addition to migraine [26,27]. Therefore, as various factors may play a role, the precise causes or triggers of headaches in children with neurodevelopmental disorders remain unclear. The present study demonstrated that comorbid neurodevelopmental disorders were associated with a higher morning headache prevalence. However, this did not emerge as a significant independent risk factor in the multiple regression analysis, possibly due to other factors such as sleep and sensory hyper-responsiveness, which may have acted as indirect risk factors.

Children with OI frequently develop headaches; however, the ICHD-3 does not list these as secondary headaches. The concept of an OI diagnosis varies considerably across countries [15,28,29,30]. Commonly accepted concepts include orthostatic hypotension (OH), orthostatic dysregulation (OD), and postural orthostatic tachycardia syndrome (POTS). OI may present with either OH and POTS or with OD. Regarding comorbid headache prevalence in patients with OI, the study by Tanaka et al. [6] reported that 68% of children with OI aged 10–16 years presented with headache, whereas Boris and Bernadzikowski [31] reported that >90% of patients with POTS and a median age of 13.1 years presented with headache. Headache has been reported to coexist with OI; however, the temporal onset of headache remains undefined [6,31]. The results of the present study indicated that orthostatic onset headache represented a significant risk factor for OI, whereas headache with an indeterminate onset timing had a lower risk for OI. In this study, vertigo and dizziness were evaluated as core symptoms of OI. Although these vestibular symptoms overlap with the diagnostic criteria for vestibular migraine of childhood, distinguishing between the two can be clinically challenging in pediatric practice. Since vestibular migraine of childhood remains in the Appendix of the ICHD-3 (ICHD-3 A1.6.6) [7], we prioritized primary headache diagnoses based on the main text of the ICHD-3. Furthermore, our analyses focused on onset timing patterns rather than detailed sub-classification of migraine types.

Although sleep disorder did not emerge as a significant independent risk factor for awakening headache in this study, research has established a strong correlation between sleep disorders and awakening headache [7,28]. Several sleep disorders have been associated with headaches, particularly obstructive sleep apnea syndrome (OSAS), which is well-documented in adults with headache as a comorbid condition [9]. The absence of an association in this study may be explained by the exclusion of patients with an OSAS diagnosis. Moreover, awakening headache has been suggested to occur with sleep disorders other than OSAS [32]. A study demonstrated that morning headache occurrence was often linked to reduced total sleep time, sleep efficiency, and rapid eye movement sleep, along with increased wake time during the preceding night [32]. Despite this, sleep disorder was not significantly associated with morning headache in the present study. This lack of correlation may have been due to the high incidence of OI coexistence and psychosocial factors with sleep disorders. Previous studies have reported an association between OI, particularly POTS, and various sleep disturbances, including difficulties with sleep onset, maintenance, duration, and quality, and issues such as excessive daytime sleepiness [28,33]. In addition, psychosocial factors may also cause sleep disturbances [34]. These findings suggest that when obstructive sleep apnea is excluded as the cause of a morning headache, the involvement of other primary sleep disorders may be less likely. Instead, coexisting OI and psychosocial factors may present a more probable underlying cause. This highlights the importance of a comprehensive evaluation of both physiological and psychosocial factors for patients with morning headaches, particularly for those without a clear sleep disorder diagnosis. Consequently, in our multivariate model, the statistical impact of sleep disorders might be accounted for by these closely interrelated comorbid conditions, rather than acting as a standalone predictor.

Recurrent headaches in children are closely correlated with psychological functioning and psychosocial factors [35,36,37]. Although environmental stress exacerbates primary headache, particularly migraine, certain personality traits may also contribute. A meta-analysis showed that adults with migraine tend to avoid harm, persevere, and demonstrate self-direction [38]. Children often exhibit temperamental behavior, inflexibility, and a tendency toward internalizing behaviors [39,40]. Patients with tension-type headache or migraine struggle with emotional expression and imagination use [41]. Despite numerous studies on the relationships between emotional and psychosocial factors and pediatric primary headache, evidence identifying specific headache characteristics indicating comorbid psychosocial factors remains limited. The present study demonstrated a higher prevalence of morning headache in individuals with comorbid psychosocial factors, with awakening headache emerging as a significant independent risk factor. These results may aid in identifying comorbid psychosocial factors in pediatric patients with migraine and tension-type headache.

However, we must consider alternative interpretations for these findings. The prevalence of morning symptom reporting may be influenced by recall bias; the patients with higher psychological distress might have more readily associated their symptoms with the time of awakening. Additionally, behavioral factors, such as anxiety related to school attendance or morning routines, could have exacerbated or highlighted the perception of headache symptoms during these hours.

We addressed potential confounding by headache severity and chronicity. In our cohort, headache frequency exhibited a significant ceiling effect, with 59.4% of patients reporting daily headaches (28 days/month). To maintain statistical robustness, we adjusted for chronicity using binary classification (daily vs. non-daily). Our findings demonstrated that awakening headache onset remained an independent predictor of psychosocial comorbidities even when daily headache occurrence was controlled.

The association between onset timing and comorbidities may be further elucidated by the role of calcitonin gene-related peptide (CGRP) in the trigeminovascular system. Recent insights suggest that circadian vulnerability and stress reactivity interact with CGRP-mediated pathways, particularly in pediatric migraine [42]. For patients with awakening headaches, sleep disruption may lower the threshold for CGRP release, triggering headaches upon waking. Furthermore, CGRP is involved in autonomic regulation; thus, its interaction with the trigeminovascular system might contribute to orthostatic mechanisms and the high prevalence of OI observed in our cohort. Integrating CGRP-targeted perspectives can provide a more robust pathophysiological understanding of how onset timing reflects distinct clinical phenotypes in pediatric headache.

This study has some limitations. First, its retrospective design prevented assessments of severity or detailed classifications of each comorbid condition. Second, several methodological constraints warrant mention: (1) the recording of headache onset timing relied on retrospective reporting by patients and guardians, which is inherently susceptible to recall bias; (2) although diagnoses were based on clinical criteria and validated questionnaires under the consistent oversight of a single physician, we did not employ more objective diagnostic tools such as polysomnography for sleep disorders or structured psychiatric diagnostic interviews for psychological evaluation; and (3) the limited sample size from a single center prevented a comprehensive investigation of primary headache types other than migraine, thus further studies with larger, multi-center cohorts are warranted. Finally, not all potential covariates related to headache onset timing were included; hence, other relevant covariates may exist.

## 5. Conclusions

Our study demonstrated that headache onset timing was a significant predictor for specific comorbid conditions in pediatric patients. Awakening headache was strongly associated with psychosocial factors, whereas orthostatic onset headache was highly predictive of OI occurrence. These findings suggest that identifying headache onset timing in clinical settings can facilitate the early detection of comorbidities, enabling a more comprehensive and tailored therapeutic approach for pediatric migraine and tension-type headache.

## Figures and Tables

**Table 1:** Patient baseline demographics and clinical characteristics.

Total Number of Patients Enrolled	106
Median age in years (range)	13 (6–17)
Sex, *n* (%)	
Males	41 (38.7)
Females	65 (61.3)
Headache classification, *n* (%)	
Migraine	90 (84.9)
Tension-type headache	16 (15.1)
Monthly headache frequency, median (range)	28 (0.5–28)
Timing of headache onset, *n* (%)	
Morning	
Awakening	35 (33.0)
Orthostatic	22 (20.8)
Midmorning	5 (4.7)
Daytime	2 (1.9)
Evening	8 (7.5)
Nighttime	1 (0.9)
Indeterminate	33 (31.1)
Comorbid condition, *n* (%)	
None	34 (32.1)
Neurodevelopmental disorder	16 (15.1)
Orthostatic intolerance	43 (40.6)
Sleep disorder	38 (35.8)
Psychosocial factors	41 (38.7)

**Table 2:** Correlations between headache onset timing and comorbid conditions.

Variables	Comorbid Condition	τ	*p*
Neurodevelopmental disorder	Orthostatic intolerance	−0.0263	0.7873
	Sleep disorder	0.0695	0.4766
	Psychosocial factors	0.1521	0.1191
Orthostatic intolerance	Sleep disorder	0.384	<0.0001
	Psychosocial factors	0.1723	0.0774
Sleep disorder	Psychosocial factors	0.2142	0.0282

**Table 3:** Multiple logistic regression analysis of comorbid conditions risks for headache onset timing.

	Neurodevelopmental Disorder	Orthostatic Intolerance	Sleep Disorder	Psychosocial Factors
Awakening, *n* (%)	6 (17.1)	20 (57.1)	18 (51.4)	22 (62.9)
OR, (95% CI)	0.79 (0.22–2.82)	1.56 (0.54–4.51)	1.57 (0.55–4.48)	2.82 (1.05–7.52)
Indeterminate, *n* (%)	3 (9.1)	4 (12.1)	5 (15.2)	9 (27.3)
OR, (95% CI)	0.54 (0.12–2.44)	0.19 (0.05–0.71)	0.44 (0.12–1.66)	1.19 (0.35–4.03)
Orthostatic, *n* (%)	4 (18.2)	17 (77.3)	10 (45.5)	6 (27.3)
OR, (95% CI)	2.73 (0.58–12.89)	5.34 (1.37–20.84)	0.55 (0.15–1.96)	0.21 (0.06–0.80)

CI, confidence interval; OR, odds ratio.

## Data Availability

The original contributions presented in this study are included in the article. Further inquiries can be directed to the corresponding author.

## Abbreviations

The following abbreviations are used in this manuscript:

OI	orthostatic intolerance
ICHD-3	International Classification of Headache Disorders, 3rd Edition
OSAS	obstructive sleep apnea syndrome
OH	orthostatic hypotension
OD	orthostatic dysregulation
POTS	postural orthostatic tachycardia syndrome
